# Index to Vols. 1 to XXIV of the Dental Cosmos

**Published:** 1884-12

**Authors:** 


### BIBLIOGRAPHICAL.

---

INDEX TO THE DENTAL COSMOS.—Vols. I. to XXIV. Inclusive. Compiled by Dr. James E. Dexter at the instance of Dr. A. L. Northrop. Publisher: The S. S. White Dental Manufacturing Co., Philadelphia. The necessity existing for a complete index to the numerous volumes of this well known and highly esteemed periodical, has long been felt, and the appearance of the presence work will be appreciated by the subscribers, and prove of great assistance for reference to the valuable subjects which have monthly appeared on its pages. We trust that the compilation may be continued in the form of successive volumes, until the index is completed so as to include the latest members. The arrangements of the present index cannot be excelled, and its compiler and instigator deserve great credit for the labor which has resulted in the present enterprise, and which cannot fail to be very acceptable to our profession.

